# Field Epidemiology and Laboratory Training Programs have been in Africa for 10 years, what is their effect on laboratory-based surveillance? Reflections from a panel at the African Society of Laboratory Medicine December 2014 Cape Town meeting

**DOI:** 10.11604/pamj.2015.20.451.6787

**Published:** 2015-04-30

**Authors:** Patrick Nguku, Fausta Mosha, Elizabeth Prentice, Tura Galgalo, Adebola Olayinka, Peter Nsubuga

**Affiliations:** 1 Nigeria Field Epidemiology and Laboratory Training Program, Nigeria; 2 Tanzania National Health Laboratory, Tanzania; 3 Medical Microbiologist, AMPATH, South Africa; 4 Kenya Field Epidemiology and Laboratory Training Program, Kenya; 5 Department of Medical Microbiology, Ahmadu Bello University Zaria, Nigeria; 6 Medical Epidemiologist, Global Public Health Solutions, Atlanta, Georgia, USA

**Keywords:** ASLM, IDSR, WHO, laboratory-based surveillance

## Opinion

In 2004 the first Field Epidemiology and Laboratory Training Program (FELTP) was piloted in Kenya and subsequently FELTPs were implemented in several African countries as a strategy to create a public health workforce that could holistically operate multi-disease surveillance and response systems, principally the World Health Organization Africa Regional Office's Integrated Disease Surveillance and Response (IDSR) system [1, 2]. Ten years later, Global Health Security has become a serious issue: Ebola in West Africa has reminded the world once again of the need to sustainably invest in public health surveillance and response systems everywhere by highlighting surveillance and response gaps in Africa, and these gaps include the lack of a public health laboratory workforce [3]. However, most discussions of the public health workforce do not include the need for public health laboratory workers and yet proper specimen collection, identification, and characterization of agents which underpins effective public health surveillance and response depends on functional public health laboratories that are operated by a competently trained workforce [4]. The authors were invited to present in a session titled “The role of FELTPs in laboratory-based surveillance” at the African Society for Laboratory Medicine (ASLM) biennial conference in December 2014 in Cape Town, South Africa. This session focused on five questions: a) what is FELTP? b) What is the state of public health laboratory-based surveillance in Africa? c) Are FELTPs contributing to public health surveillance and response in Africa? d) What challenges are FELTPs facing in implementation? The following is a summary of the presentations and discussions.

**What is FELTP?** FELTP is an adaptation of the Field Epidemiology Training Program (FETP) which started in the 1980s as an “exportation” of the United States Centers for Disease Control and Prevention (CDC) flagship training program, the 2-year postgraduate Epidemic Intelligence Service (EIS) internationally, beginning with Canada and Thailand then spreading to over 50 countries [5], with CDC support. FETPs like EIS are competency-based public health training programs, meaning that they provide a cluster for related knowledge, skills, and attitudes that enable participants to perform a variety of epidemiologic tasks in the actual work setting. However FELTPs are an modification of the FETPs in three principal ways: a) they focus on developing a workforce to holistically operate public health surveillance and response systems by including laboratory professionals and veterinarians; b) they are cognizant of the need to partner with academic institutions (especially in Africa) so that the graduates obtain relevant qualifications that are acceptable by the host country's civil service enabling them to be promoted as leaders in the government's public health workforce; and c) they focus on a set of critical outcomes in the public health system that will eventually transform public health surveillance and response systems in the host country after 5 to 10 years of continuous FELTP implementation. In a country with a successfully implemented FELTP the following critical outcomes should occur: a) functional and robust public health surveillance systems, beginning with communicable diseases and eventually adding non-communicable diseases; b) timely and effective response to public health emergencies (including outbreaks); c) a culture of evidence-based decision making in public health; d) a strengthened public health workforce (comprising leaders who are graduates of the 2-year courses and frontline implementers who are graduates of the associated competency-based short courses); e) the FELTP would have a demonstrable contribution to reduction in morbidity and mortality from priority diseases; and f) there would be networking and communication within the country (e.g., between the epidemiology and laboratory services) and between country FELTP programs. In effect if an African FELTP is working as designed then the host country would have a robust IDSR system, and the so called “left shift of the usual epidemic curve” would be evident (i.e. earlier detection of suspected outbreaks, followed by earlier confirmation from the laboratory, and earlier effective public health response, would lead to fewer cases and fewer deaths thereby shifting the usual epidemic curve to the left).

**What is the state of public health laboratories and networks in Africa?** The Public Health Laboratory Network (PHLN) is a collaborative group of laboratories, which have expertise and provide services in public health. The network aims at detecting and monitoring health events in the population. For the PHLN to be efficient, it requires a successful detection, characterization, and tracing of disease transmission that is essential for the prevention and control of public health events [6]. Public health laboratory services in Africa are characterized by the following: a) inadequate staffing both in the number of staff and the skills of the available staff, meaning there are very few properly trained laboratory workers; b) shortages and unavailability of laboratory supplies both in quantity and quality; c) shortages in laboratory equipment and the equipment that is available is poorly serviced which result in regular breakdown and frequent interruption of services; d) challenges in developing robust systems and training the workforce to operate public health laboratory networks [7]. Public health laboratories are necessary for proper implementation of the revised International Health Regulations (IHR), however, public health laboratories have almost all but disappeared in several African countries mainly because they have either been integrated with or replaced by clinical diagnostic laboratories [8]. Where public health laboratories exist they are usually only at the national level and there are few viable PHLNs. As viable PHLNs in Africa are needed for effective control and prevention of communicable diseases, it is important to develop public health laboratory workforce capacity with competencies in field epidemiology, invest in personnel and equipment for national PHLNs, develop national laboratory strategic plans and policies, establish public-private partnerships, and ensure effective laboratory leadership and funding commitment from governments [4].

**What is public health laboratory-based surveillance?** According to Eylenbosch and Noah, public health laboratory-based surveillance can be defined as the continuous analysis, interpretation, and feedback of systematically collected laboratory data, generally using methods distinguished by their practicability, uniformity and rapidity, rather than by accuracy and completeness [9]. Effective public health laboratory-based surveillance enables rapid confirmation of cases from suspected outbreaks, characterization of the isolated pathogens, and provides a reporting infrastructure in line with local, national, and international implementation of IDSR and IHR. Public health laboratory-based surveillance requires laboratory facilities and capabilities, which may be tiered if the laboratories are in a public health laboratory network. However the results of the public health laboratory-based surveillance system are only as good as the system itself and only provide a tip of the iceberg view at best as only cases that were tested in the laboratory and obtained a positive result and were subsequently reported are detected by the laboratory-based surveillance system. Public health laboratory practitioners identify three distinct phases of the public health laboratory-based surveillance framework: a) pre-analytic which encompasses all the activities that occur before the specimen reaches the laboratory (i.e. proper case diagnosis and preparation, specimen collection, handling, and safe transportation); b) analytic which are activities within the laboratory (i.e. sample reception and handling, testing, and results) and c) post analytic which is reporting laboratory results back to the place of original diagnosis and to relevant public health officials [10]. Laboratory professionals working alone answer a different public health surveillance question from epidemiologists and this answer is only obtainable from specimens that make it into a properly equipped well-functioning laboratory in the analytic phase of the laboratory-based surveillance framework. Conversely epidemiologists working alone can only contribute answers in the pre-analytic (i.e. identification of suspected outbreaks and collecting and shipping specimens) and the post-analytic phase (i.e., using reports from the laboratory to initiate public health actions) of the public health laboratory-based surveillance framework. Integration between epidemiologists and public health laboratory professionals provides a holistic answer to public health surveillance and response questions.

**Are FELTPs working as designed 10 years after the first FELTP in Kenya?** FELTPs have enrolled >1500 health workers since they were first initiated in Africa and have supported IDSR and IHR and the trainees and graduates have responded to numerous public health emergencies caused by communicable and non-communicable conditions [11–13]. Trainees have conducted several operational research studies that have provided evidence for public health decision-making including in public laboratories. Graduates and trainees are networked through the African Field Epidemiology Network, which is a service and networking alliance that was formed 2 years after the first FELTP was initiated [14], and have collaborated in several cross-border and inter-country collaborations to solve public health problems (e.g., Rift Valley Fever outbreak response collaboration between the Kenya and Tanzania FELTP) [15]. FELTPs have responded to major public health emergencies, for example at the time of the 2014 ASLM meeting the Nigeria FELTP had just completed participating in the EVD outbreak in Nigeria with 100 trainees, graduates and staff [16]. The EVD outbreak in Nigeria was controlled in just over 90 days but was still raging primarily in countries without FELTPs [17]. Specifically for the public health laboratory arena, graduates of the FELTP laboratory track currently lead several public health laboratory systems in Africa. FELTP has contributed to enhancing the public health laboratory strengthening component in ministries of health, has led to improved diagnostic and monitoring services, and is supporting the implementation of laboratory quality systems and the initiation of laboratory accreditation processes [18].

**What challenges remain, how will the next 10 years of FELTP look?** In our view, FELTP as a concept has achieved several of the outcomes that were intended but at least five key challenges remain that need to be addressed to ensure that FELTP continues from strength to strength: 1) all competency-based training programs are expensive by design and continued government commitment and funding is necessary. Unfortunately public health surveillance often suffers from a lack of funding until it is needed- the current amount of funding going to Ebola control could maintain several FELTPs for a long time and they would address several public health conditions; 2) Public health laboratory graduates continue to require a specific scheme of service within the health sector, this challenge requires involvement of non-health sector stakeholders in (e.g. the ministries of finance and public service) to be solved. Related to this, public health surveillance systems including the workforce continue to suffer from lack of funding, the time may have come for governments to consider new ways of funding public health surveillance systems given the opportunity cost of a missed outbreak. Private sector or market oriented funding may allow for functional systems that are operated by motivated staff; 3) The existing laboratory sector in general is inadequately resourced and this affects the quality of hands on practical training for the laboratory scientists in FELTP due to lack of reagents, equipment, and poor infrastructure; however increasingly donors are creating specific laboratory strengthening projects like the World Bank's East African Public Health Laboratory Networking Project that may alleviate this challenge [19]; 4) the laboratory track of FELTP depends on functional public health laboratory networks and these require laboratory policies, strategic plans, and capital-heavy investments to build and maintain laboratories, which is a medium term challenge that all governments have to embrace; 5) the FELTP paradigm as an adaptation of the original EIS/FETP model continues to be challenged by purists who believe that the original non-degree granting training for only epidemiologists, which began in the 1950s, is the only way to go. However the adaptations and trade-offs that were made when FELTP was designed to specifically address the necessities of African multi-disease surveillance and response system development which requires laboratory participation (and other cadres e.g., veterinary and environment for “one health”) and needs university engagement to produce public health leaders that can enter the civil service of their host country with new postgraduate qualifications, is likely to prove to be more relevant to modern public health challenges particularly in Africa, but this still remains an important challenge.
